# 伊沙佐米、来那度胺、地塞米松治疗复发/难治多发性骨髓瘤的疗效与安全性分析：一项国内多中心真实世界研究

**DOI:** 10.3760/cma.j.issn.0253-2727.2021.08.003

**Published:** 2021-08

**Authors:** 扬 杨, 忠军 夏, 文皓 张, 琤琤 傅, 立 鲍, 兵 陈, 凯阳 丁, 思力 王, 军 罗, 炳宗 李, 罗明 化, 威 杨, 新 周, 亮 王, 天虹 徐, 维达 王, 国林 吴, 耘 黄, 晶 李, 澎 刘

**Affiliations:** 1 复旦大学附属中山医院血液科，上海 200032 Department of Hematology, Zhongshan Hospital, Fudan University, Shanghai 200032, China; 2 中山大学肿瘤防治中心血液肿瘤科，广州 510060 Department of Hematologic Oncology, Sun Yat-sen University Cancer Center, Guangzhou 510060, China; 3 上海交通大学医学院附属新华医院血液科 200092 Department of Hematology, Xinhua Hospital Affiliated to Shanghai Jiaotong University School of Medicine, Shanghai 200092, China; 4 苏州大学附属第一医院血液科，江苏省血液研究所 210056 Department of Hematology, The First Affiliated Hospital of Soochow University, Jiangsu Institute of Hematology, Suzhou 210056, China; 5 北京积水潭医院血液科 100035 Department of Hematology, Beijing Jishuitan Hospital, Beijing 100035, China; 6 南京医科大学附属鼓楼医院血液科 210008 Department of Hematology, The Affiliated Nanjing Drum Tower Hospital, Nanjing University Medical School, Nanjing 210008, China; 7 安徽省肿瘤医院血液科，合肥 230031 Department of Hematology, Anhui Provincial Cancer Hospital, Hefei 230031, China; 8 厦门大学附属第一医院血液科 361003 Department of Hematology, The First Affiliated Hospital of Xiamen University, Xiamen 361003, China; 9 广西医科大学第一附属医院血液科，南宁 530021 Department of Hematology, The First Affiliated Hospital of Guangxi Medical University, Nanning 530021, China; 10 苏州大学附属第二医院血液科 215004 Department of Hematology, The Second Affiliated Hospital of Soochow University, Suzhou 215004, China; 11 河北大学附属医院血液科，保定 071030 Department of Hematology, The Affiliated Hospital of Hebei University, Baoding 071030, China; 12 中国医科大学盛京医院血液科，沈阳 110004 Department of Hematology, Shengjing Hospital of China Medical University, Shenyang 110004, China; 13 无锡市人民医院血液科 214023 Department of Hematology, Wuxi People Hospital, Wuxi 214023, China; 14 南方医科大学珠江医院血液科，广州 510280 Department of Hematology, Zhujiang Hospital of Southern Medical University, Guangzhou 510280, China

**Keywords:** 多发性骨髓瘤, 伊沙佐米, 来那度胺, 疗效, 安全性, Multiple myeloma, Ixazomib, Lenalidomide, Treatment outcome, Safety

## Abstract

**目的:**

评估伊沙佐米、来那度胺、地塞米松（IRd）方案治疗复发/难治多发性骨髓瘤（MM）的疗效及安全性。

**方法:**

纳入2018年7月至2020年5月于国内14个中心接受至少1个周期IRd方案治疗的复发/难治MM患者。除7例起始剂量作调整的患者以外，其余患者伊沙佐米起始剂量为4 mg。来那度胺剂量根据患者肌酐清除率进行调整。每周期进行疗效及安全性评估。

**结果:**

共纳入74例患者，中位年龄65岁，11例（14.9％）患者曾接受>三线治疗。总体反应率（ORR）为54.1％（40/74），其中7例（9.5％）达严格意义的完全缓解或完全缓解，14例（18.9％）达非常好的部分缓解，19例（25.7％）达部分缓解，中位无进展生存时间为9.9月，中位总生存时间为20个月，中位获得反应时间为1个月。总体疗效与生存结果与TOURMALINE-MM1中国延展性研究（TOURMALINE-MM1 CCS）相近。硼替佐米难治、来那度胺难治、两药均难治患者的ORR分别为52.0％（13/25）、57.1％（4/7）和33.3％（6/18）。3～4级不良事件（AE）发生率为36.5％（27/74）。主要血液学不良反应为贫血、血小板减少、淋巴细胞减少及中性粒细胞减少。主要非血液学不良反应为乏力、消化道症状及感染。2例患者出现3级周围神经病变。与不符合TOURMALINE-MM1 CCS试验入组标准的患者相比，符合入组标准的患者ORR更高［77.8％（14/18）对46.4％（26/56），*P*＝0.020］，两组3～4级AE发生率的差异无统计学意义［33.3％（6/18）对37.5％（21/56），*P*＝0.749］。

**结论:**

IRd方案治疗复发/难治MM具有较好的疗效及安全性。

## Abstract (en)

**Objective:**

To evaluate the efficacy and safety profile of ixazomib/lenalidomide/dexamethasone (IRd) in Chinese patients with relapsed/refractory multiple myeloma (MM).

**Methods:**

This study comprising 14 medical centers in China included patients with relapsed/refractory MM who received at least. Ixazomib at an initial oral dose of 4 mg was administered. Seven patients had dose adjustment to 3 mg at the time of first dose. The lenalidomide doses were adjusted according to creatinine clearance rate. The efficacy and safety were evaluated every cycle.

**Results:**

In the study cohort of 74 patients, the median age was 65 years and 11 (14.9％) patients received over three lines of therapy. Overall response rate (ORR) was 54.1％ (40/74), and 7 (9.5％), 14 (18.9％), and 19 (25.7％) patients achieved stringent complete response or complete response, very good partial response, and partial response, respectively. The median progression-free survival and overall survival were 9.9 and 20 months, respectively. The median time to response was 1 month. The efficacy and survival outcome were similar to those reported in the Tourmaline-MM1 China Continuous Study. The ORR of patients refractory to bortezomib, lenalidomide, and bortezomib plus lenalidomide were 52.0％ (13/25), 57.1％ (4/7), and 33.3％ (6/18), respectively. The rate of grade 3-4 adverse events was 36.5％ (27/74). Common hematological toxicities were anemia, thrombocytopenia, lymphopenia, and neutropenia. Common non-hematological toxicities were fatigue, gastrointestinal symptoms, and infections. Two cases of grade 3 peripheral neuropathy were reported. The patients eligible for the Tourmaline-MM1 China Continuous Study had a higher ORR than the ineligible patients [77.8％ (14/18) *vs* 46.4％ (26/56), *P*＝0.020]. There was no difference in the rate of grade 3–4 adverse events [33.3％ (6/18) *vs* 37.5％ (21/56), *P*＝0.749].

**Conclusion:**

The IRd regimen had good efficacy and acceptable toxicity in Chinese patients with relapsed/refractory MM.

近年来，随着新药的研发及广泛应用，多发性骨髓瘤（MM）患者的总体预后得到了明显改善[1]。伊沙佐米是首个口服蛋白酶体抑制剂。在全球Ⅲ期临床研究TOURMALINE-MM1及中国延展性研究（TOURMALINE-MM1 CCS）中，伊沙佐米+来那度胺+地塞米松（IRd）诱导方案对复发/难治MM显示出了较好的疗效及安全性[2]–[3]。然而，IRd方案在中国复发/难治MM患者中的真实疗效及安全性仍缺乏来自大样本、多中心的数据。本研究分析了国内14个中心74例接受IRd诱导方案的复发/难治MM患者，以评价该治疗方案在国内临床实践中的疗效及安全性。

## 病例与方法

1. 病例：纳入2018年7月至2020年5月于国内14个中心就诊并接受至少1个周期IRd方案诱导治疗的74例复发/难治MM患者，所有患者均符合活动性MM诊断标准[4]，且符合国际骨髓瘤工作组（IMWG）及美国血液协会相关共识对“复发/难治”的定义[5]–[6]。主要排除标准为：接受IRd方案治疗不足1个周期患者。根据患者基线临床数据和既往用药，18例（24.3％）符合TOURMALINE-MM1及Tourmaline-MM1 CCS（NCT01564537）临床试验纳入标准[2]–[7]，56例（75.7％）不符合。不符合标准的主要原因为：①既往治疗线数>三线；②蛋白酶体抑制剂类药物或来那度胺难治；③美国东部肿瘤协作组（ECOG）评分> 2分；④肾功能不全（定义为肌酐清除率< 30 ml/min）；⑤合并不稳定的心血管疾病；⑥合并其他恶性肿瘤；⑦合并淀粉样变性；⑧中性粒细胞计数< 1.0 × 10^9^/L或PLT < 75×10^9^/L；⑨中枢神经系统受累。采集患者的临床数据，包括年龄、性别、ECOG评分、血常规、肝肾功能、电解质、血清蛋白电泳、血/尿免疫固定电泳、血游离轻链、影像学检查（X射线、CT、PET-CT等）、病理检查、细胞遗传学检查等。高危细胞遗传学定义为间期荧光原位杂交或中期细胞遗传学检查检测到del（17p）、t（4;14）或t（14;16）[8]。

2. 治疗方案：患者接受IRd方案治疗，具体为：伊沙佐米4 mg，口服，第1、8、15天；来那度胺25 mg，口服，第1～21天；地塞米松40 mg，口服，第1、8、15、22天；每28 d为一个周期。7例患者的伊沙佐米起始剂量调整为3 mg。来那度胺剂量根据患者肌酐清除率进行调整。

3. 疗效评价：治疗前和每周期治疗结束后，采集评估疗效和安全性所需的血液样本和24 h尿液样本。根据IMWG制定的疗效评估标准对患者进行疗效评估，分为严格意义的完全缓解（sCR）、完全缓解（CR）、非常好的部分缓解（VGPR）、部分缓解（PR）、微小缓解（MR）、疾病稳定（SD）和疾病进展（PD）[9]。sCR率、CR率、VGPR率及PR率之和为总有效率（ORR）。

4. 安全性评价：根据美国国家癌症研究所常见毒性标准5.0版，每周期对患者用药发生的不良事件（AE）进行分级[10]。

5. 随访：通过查阅病历或电话的方式进行随访，随访截止日期为2020年9月9日。总生存（OS）时间定义为从首次使用伊沙佐米至死亡或随访终止时间。无进展生存（PFS）时间定义为从首次使用伊沙佐米至疾病进展、死亡或随访终止时间。

6. 统计学处理：应用SPSS 20.0软件进行统计学分析。计量资料的组间比较采用*t*检验，当组内计量资料不符合正态分布或组间计量资料方差不齐时采用Wilcoxon秩和检验，分类变量的组间比较采用*χ*^2^检验，当不满足*χ*^2^检验时采用Fisher检验，生存率的比较采用Log-rank检验，生存曲线绘制采用Kaplan-Meier法。*P* < 0.05为差异有统计学意义。

## 结果

1. 患者的临床特征：患者的中位年龄为65（33～87）岁，45.2％为ISS Ⅲ期患者，在进行细胞遗传学检查的47例患者中，12例（25.5％）为高危患者，35例（74.5％）为标危患者。14.9％患者曾接受>三线治疗。根据TOURMALINE-MM1及TOURMALINE-MM1 CCS临床试验的入组条件，将74例患者分为符合MM1 CCS试验组及不符合MM1 CCS试验组（表1）。与符合MM1 CCS试验组相比，不符合MM1 CCS试验组≥75岁、ISS Ⅲ期、ECOG评分≥2分及贫血患者比例较高，但两组的差异无统计学意义（表1）。

**表1:** 符合及不符合MM1 CCS试验组患者的临床特征比较

临床特征	符合MM1CCS试验组（18例）	不符合MM1CCS试验组（56例）	*χ*^2^值或*t*值	*P*值
中位年龄［岁，*M*（范围）］	64.5（33～78）	65.0（42～87）	−1.741	0.086
年龄［例（％）］			1.457	0.483
18～64岁	10（55.6）	32（57.1）		
65～74岁	7（38.9）	16（28.6）		
≥75岁	1（5.6）	8（14.3）		
男性［例，（％）］	10（55.6）	34（60.7）	0.150	0.698
伊沙佐米治疗前ISS分期［例（％）］			2.532	0.282
Ⅰ期	7（41.2）	14（25.0）		
Ⅱ期	5（29.4）	14（25.0）		
Ⅲ期	5（29.4）	28（50.0）		
高危细胞遗传学［例（％）］	2（28.6）	10（25.0）	0.000	1.000
ECOG评分≥2分［例（％）］	3（16.7）	20（35.7）	2.307	0.129
高钙血症［例（％）］	0（0）	3（5.4）	–	1.000
血肌酐> 177 µmol/L［例（％）］	0（0）	8（14.3）	1.460	0.227
贫血［例（％）］	5（29.4）	27（48.2）	1.873	0.171
骨损害［例（％）］	17（94.4）	45（80.4）	1.088	0.297
髓外浆细胞瘤［例（％）］	6（33.3）	12（21.4）	0.502	0.479
周围神经病变［例（％）］	4（22.2）	10（17.9）	0.004	0.948
先前接受治疗线数［例（％）］			6.979	0.079
一线	7（38.9）	16（28.6）		
二线	6（33.3）	16（28.6）		
三线	5（27.8）	13（23.2）		
≥四线	0（0）	11（19.6）		
接受硼替佐米治疗［例（％）］	16（88.9）	53（94.6）	0.094	0.759
硼替佐米难治［例（％）］	0（0）	43（76.8）	32.993	< 0.001
接受来那度胺治疗［例（％）］	9（50.0）	32（57.1）	0.281	0.596
来那度胺难治［例（％）］	0（0）	25（44.6）	12.136	< 0.001
接受沙利度胺治疗［例（％）］	8（44.4）	26（46.4）	0.022	0.883
沙利度胺难治［例（％）］	3（16.7）	23（41.1）	3.560	0.059

注：MM1 CCS：TOURMALINE-MM1中国延展性研究；ECOG：美国东部肿瘤协作组；−：采用Fisher检验，无统计量

不符合MM1 CCS试验标准的最常见原因为蛋白酶体抑制剂类药物（均为硼替佐米）难治（43例）和来那度胺难治（25例）。11例患者既往治疗线数>三线（四线7例，五、六线各2例）；10例患者ECOG评分>2分；8例患者肾功能不全；4例患者合并淀粉样变性；3例患者PLT < 75×10^9^/L。另有因不稳定的心血管疾病及中枢神经系统疾病未被纳入临床试验的患者各1例。每例患者至少存在一种不符合MM1 CCS试验标准的原因。

2. 疗效和生存分析：在74例接受IRd方案的患者中，7例患者伊沙佐米起始剂量调整为3 mg，其余患者起始剂量为4 mg，治疗期间无患者需减量。来那度胺剂量根据患者肌酐清除率进行调整。接受IRd方案的患者中位治疗4（1～28）个周期。至研究截止时，29例（39.1％）患者仍在治疗。45例患者终止治疗的原因如下：19例（25.7％）患者PD，10例（13.5％）患者不能耐受不良反应，16例（21.6％）患者为其他原因。

截至随访结束，中位随访9.2（1.0～26.4）个月。74例患者的ORR为54.1％（40/74），其中达sCR或CR者7例，达VGPR者14例，达PR者19例，达到最佳疗效的中位时间为1.0（0.7～17.5）个月。符合MM1 CCS试验组患者的ORR高于不符合MM1 CCS试验组，差异有统计学意义（表2）。12例高危患者的ORR与35例低危患者相比，差异无统计学意义（50.0％对51.4％，*P*＝0.932）。

**表2:** 符合及不符合MM1 CCS试验组患者的疗效比较

疗效	符合MM1CCS试验组（18例）	不符合MM1CCS试验组（56例）	*χ*^2^值或*Z*值	*P*值
ORR［例（％）］	14（77.8）	26（46.4）	5.390	0.020
≥VGPR率［例（％）］	9（50.0）	12（21.4）	5.471	0.019
最佳疗效［例（％）］			11.814	0.037
CR+sCR	5（27.8）	2（3.6）		
VGPR	4（22.2）	10（17.9）		
PR	5（27.8）	14（25.0）		
MR	1（5.6）	6（10.7）		
SD	3（16.7）	19（33.9）		
PD	0（0）	5（8.9）		
达到最佳疗效时间［月，*M*（范围）］	1.8（0.7～7.7）	1.0（0.9～17.5）	−0.985	0.324
PFS时间［月，*M*（95％*CI*）］	NR	7.5（4.3～10.6）	2.976	0.085
OS时间［月，*M*（95％*CI*）］	NR	20.0（14.2～25.9）	2.911	0.088
伊沙佐米治疗周期数［个，*M*（范围）］	4（1～16）	4（1～28）	−0.497	0.619

注：MM1 CCS：TOURMALINE-MM1中国延展性研究；ORR：总有效率；VGPR：非常好的部分缓解；CR：完全缓解；sCR：严格意义的完全缓解；PR：部分缓解；MR：微小缓解；SD：疾病稳定；PD：疾病进展；PFS：无进展生存；OS：总生存；NR：未达到

所有患者的中位PFS时间为9.9（95％ *CI* 5.5～14.4）个月，中位OS时间为20.0（95％ *CI* 14.6～25.4）个月，伊沙佐米中位治疗4（1～28）个周期。根据年龄（18～64岁对65～74岁对≥75岁）、ECOG评分（0～1分对≥2分）、既往治疗线数（一线治疗对>一线治疗）及细胞遗传学危险度（标危对高危）分组，PFS和OS的差异均无统计学意义（PFS的*P*值分别为0.950、0.410、0.310、0.250；OS的*P*值分别为0.680、0.970、0.510、0.090）。符合MM1 CCS试验组的PFS、OS均优于不符合MM1 CCS试验组，但差异均无统计学意义（表2）。高危、标危患者的中位PFS时间分别为7.0个月与11.8个月，但差异无统计学意义（*P*＝0.245）；高危、标危组患者均未达到中位OS时间，12个月OS率分别为78.8％和77.1％（*P*＝0.903）。硼替佐米难治、来那度胺难治及两药均难治患者的疗效见表3。

**表3:** 硼替佐米难治、来那度胺难治及两药均难治患者的疗效

疗效	仅硼替佐米难治组（25例）	仅来那度胺难治组（7例）	硼替佐米、来那度胺难治组（18例）
ORR［例（％）］	13（52.0）	4（57.1）	6（33.3）
≥VGPR［例（％）］	7（28.0）	1（14.3）	3（16.7）
最佳疗效［例（％）］			
CR+sCR	1（4.0）	0（0）	1（5.6）
VGPR	6（24.0）	1（14.3）	2（11.1）
PR	6（24.0）	3（42.9）	3（16.7）
MR	3（12.0）	1（14.3）	2（11.1）
SD	7（28.0）	2（28.6）	8（44.4）
PD	2（8.0）	0（0）	2（11.1）
达到最佳疗效时间［月，*M*（范围）］	1.0（0.9～17.5）	1.0（0.9～10.0）	1.0（0.9～5.1）
PFS时间［月，*M*（95％*CI*）］	NR	NR	4.8（2.0～7.6）
OS时间［月，*M*（95％*CI*）］	17.0（13.7～20.3）	NR	20.0（NA）
伊沙佐米治疗周期数［个，*M*（范围）］	4.0（1～28）	4.0（1～5）	4.5（1～13）

注：ORR：总有效率；VGPR：非常好的部分缓解；CR：完全缓解；sCR：严格意义的完全缓解；PR：部分缓解；MR：微小缓解；SD：疾病稳定；PD：疾病进展；PFS：无进展生存；OS：总生存；NR：未达到；NA：无数据

3. 安全性：所有患者均接受安全性评估。最常见的血液学AE为贫血（29例，39.2％）、血小板减少（28例，37.8％）、淋巴细胞减少（26例，35.1％）和中性粒细胞减少（22例，29.7％）。15例患者发生3～4级AE，其中血小板减少最常见（5例，6.8％）。常见非血液学AE为乏力（18例，24.3％）、腹泻（17例，31.5％）及感染（17例，31.5％）。感染患者中肺部感染11例，上呼吸道感染4例，肠道感染、牙周脓肿各1例。乏力和腹泻基本为1～2级。12例患者发生3～4级感染，需要住院治疗，其中肺部感染9例，上呼吸道感染2例，肠道感染1例。13例患者发生周围神经病变（17.6％），2例为3级。

AE发生率为98.6％（73/74），3～4级AE发生率为36.5％（27/74）。9例患者因3～4级AE停药，原因为腹泻（3例）、心力衰竭（2例，均合并心脏淀粉样变性）、血液学毒性（1例）、周围感觉神经病变（1例）、肺部感染（1例）、严重恶心呕吐（1例）。另有1例患者因无法耐受2级周围感觉神经病变停药。符合MM1 CCS试验组AE发生率为100％（18/18），不符合MM1 CCS试验组AE发生率为98.2％（55/56），两组的差异无统计学意义（*P*＝1.000）。不符合MM1 CCS试验组3～4级AE发生率为37.5％，与符合MM1 CCS试验组相近（33.3％），差异无统计学意义（*P*＝0.102）。符合MM1 CCS试验组因AE停药发生率22.2％（2/18），不符合MM1 CCS试验组因AE停药发生率14.2％（8/56），两组的差异无统计学意义（*P*＝1.000）。未出现治疗过程中死亡病例。

## 讨论

本研究中，74例复发/难治MM患者接受IRd方案诱导治疗，获得最佳疗效的中位时间为1个月，ORR为54.1％，中位OS时间为20个月，中位PFS时间9.9个月，反应率与国内既往小样本单中心回顾性研究报道相似[9]–[10]。

真实世界研究与临床试验结果存在差别的原因之一是患者基线水平存在差异[11]。多项伊沙佐米真实世界研究显示ORR为66.9％～73.8％，中位PFS时间为16.6～27.6个月，与TOURMALINE-MM1结果较为一致[12]–[17]。而匈牙利和日本的研究中纳入多次治疗后复发/难治MM患者，中位PFS时间分别为11.4个月及11.9个月，明显劣于临床试验结果[18]–[19]。TOURMALINE-MM1 CCS是独立于TOURMALINE-MM1、基于中国人群的大型Ⅲ期临床研究。在MM1 CCS试验中，IRd组患者的ORR为56％，≥VGPR率25％，中位PFS时间为6.7个月，中位OS时间为25.8个月[3]。本研究与MM1 CCS试验IRd方案组疗效基本一致，但我们纳入了更多ISS Ⅲ期、既往接受大于三线治疗、ECOG评分≥2分的患者。在不符合MM1 CCS入组标准的患者中，≥75岁、ISS Ⅲ期、ECOG评分≥2分和贫血患者比例偏高。18例符合MM1 CCS入组标准患者的ORR高达77.8％，≥VGPR率为50％，中位PFS、OS时间均未达到，明显优于不符合入组标准的患者及MM1 CCS试验IRd方案组患者。需要指出的是，仅3例（16.6％）患者沙利度胺难治，而MM1 CCS试验的IRd方案组中65％患者沙利度胺难治。上述结果进一步提示了基线水平差异对研究结果的影响[11],[20]–[21]。

本研究的亮点之一是纳入了被MM1 CCS及MM1试验排除的硼替佐米和来那度胺难治患者。来自英国的两项真实世界研究表明，蛋白酶体抑制剂类药物难治患者接受IRd方案获益有限，但部分患者曾接受卡非佐米治疗，或未详细说明难治的药物名称[12],[16]。一项日本研究认为硼替佐米难治不影响IRd方案的疗效及患者生存，而来那度胺难治患者接受IRd方案治疗获益有限[19]。本研究中，仅硼替佐米难治患者的ORR与总体患者相似，而25例来那度胺难治患者的ORR为40.0％，其中18例硼替佐米、来那度胺两药均难治患者的ORR仅33.3％，提示硼替佐米难治患者能够从伊沙佐米的治疗中获益，而来那度胺难治患者需要寻求更为有效的方案[9]。仅来那度胺难治患者7例，信息有限，未单独进行分析。

随着新药的研发和广泛运用，MM患者的预后已得到显著改善，但高危细胞遗传学患者的预后依然不佳[22]。本研究中，高危组患者的ORR与标危组相当，两组中位PFS时间的差异无统计学意义。MM1研究结果提示伊沙佐米可部分克服高危细胞遗传学的不良预后，但此研究中del（17p）的临界值为5％，是否存在预后意义尚无定论[23]。此外，有文献提出同类药物硼替佐米持续治疗能部分克服不良细胞遗传学的作用[24]。需要指出的是，本队列中各中心所采用的检测方法不同，且存在部分细胞遗传学资料缺失的情况，统计学结果应谨慎解读。

值得注意的是，部分患者使用伊沙佐米无法进一步降低肿瘤负荷，但能够使病情稳定或减缓肿瘤进展速度，长期使用亦有一定获益。本研究7例患者在IRd方案治疗4个周期评估为SD后继续采用原方案，其中2例患者分别在第5、10个周期后评估为VGPR，3例患者PD，其余2例为持续SD且仍在治疗中。另有3例患者在第1或第2个周期评估为PD，但继续原方案治疗，后续疾病呈缓慢进展状态。一项分析TOURMALINE-MM3的研究显示，对于维持治疗中缓解程度未进一步改善的初治MM患者，伊沙佐米能维持SD状态，进而导致PFS获益[25]。这提示在国内新药选择有限的情况下，使用包含伊沙佐米在内的全口服方案能使一部分患者的疾病处于稳定状态，是复发/难治MM患者的可选治疗方案。

在既往研究中，伊沙佐米的安全性得到了良好证实。IRd方案最常见的AE包括：血小板减少、皮疹、腹泻、呕吐等，其中3～4级AE中血小板减少约占10％[2]。若干真实世界研究中AE的发生率和种类与MM1研究的报道较为一致，且多线治疗患者AE发生率未见明显升高[13]。本研究中，血液学AE主要为贫血、血小板减少、淋巴细胞减少和中性粒细胞减少，3～4级血液学AE共15例（20.3％）。非血液学AE主要为乏力、消化道症状及感染。周围神经病变共发生13例（17.6％），2例为3～4级，其发生率较第一代蛋白酶体抑制剂硼替佐米低，后者周围神经病变发生率约为40％[26]。治疗过程中无患者出现带状疱疹。受限于回顾性资料分析，本研究各级AE发生率较MM1 CCS研究低，但伊沙佐米的总体安全性良好。符合MM1 CCS试验组3～4级AE的发生率及停药率与不符合MM1 CCS试验组相近。

本研究确认了IRd方案治疗复发/难治MM的有效性与安全性。符合临床试验入组条件的患者表现出更好的疗效、生存情况，3～4级AE发生率与不符合临床试验入组条件患者相似。
